# Timing of isolation from an enriched environment determines the level of aggressive behavior and sexual maturity in Siamese fighting fish (*Betta splendens*)

**DOI:** 10.1186/s40850-021-00081-x

**Published:** 2021-05-10

**Authors:** Eri Iwata, Kyouhei Masamoto, Hiroyuki Kuga, Miho Ogino

**Affiliations:** 1 Department of Science and Technology, Iwaki Meisei University, 5-5-1 Chuoudai, Ihino, Iwaki, Fukushima, 970-8551 Japan; 2 Institute of Osaka Marine Research, 19-34, Shimodachou, Nishi Ku, Sakai City, Osaka 593-8329 Japan; 3 Faculty of Veterinary Medicine, Okayama University of Science, 1-3, Ikoinooka, Imabari City, Ehime 794-8555 Japan

**Keywords:** Environmental enrichment, Fighting fish, *Betta splendens*, Aggression, Maturity

## Abstract

**Background:**

Teleost fish are known to respond to environmental manipulation, which makes them an ideal model animal for testing relationships between the environment and behavior. The Siamese fighting fish, *Betta splendens*, is a solitary, highly territorial fish that displays fierce stereotyped aggressive behavior toward conspecifics or members of other species. Adult fish, especially males, are generally housed in isolation in captivity. Here we report evidence that an enriched rearing environment can decrease the level of aggression in bettas and enable adults to be housed in groups.

**Results:**

*B. splendens* individuals were hatched in our laboratory and raised in groups in an enriched environment. At the juvenile or subadult stage, some individuals were relocated to a poor environment and kept in isolation. To evaluate aggression, a mirror-image test was conducted at the juvenile, subadult, and adult stages for each fish, and body parameters as well as plasma concentrations of 11-ketotestosterone, estradiol, and cortisol were evaluated. Male and female adult bettas raised in a group showed lower levels of aggression than other adult fish. The magnitude of threatening behavior was greater in adult bettas isolated as subadults, whereas the magnitude of fighting behavior was grater in adult bettas isolated as juveniles. The influence of rearing conditions on behavior was greater in females than in males. Plasma cortisol concentrations of adult bettas isolated as subadults after the mirror-image test were higher than those in other experimental groups. Adult males isolated as subadults had significantly higher plasma concentrations of 11-ketotestosterone than males raised in a group and isolated as juveniles. Females isolated as subadults had a higher gonadosomatic index than females raised in a group and females isolated as juveniles.

**Conclusions:**

These results indicate that bettas can be kept in a group under enriched environments and that the timing of isolation influences the aggression and sexual maturity of bettas. Female and male bettas responded differently to environmental manipulation. Judging from their level of sexual maturity, bettas isolated as subadults show proper development.

## Background

*Betta splendens* (family Anabantidae) is native to still freshwater areas in Thailand, Malaysia, Cambodia, and Myanmar. The fish, which is highly territorial in nature, was initially domesticated for fighting in Thailand [1], and the natural history of bettas is not well known. Their natural habitat is characterized by heavy emergent vegetation and shallow water, such as muddy bottoms or flooded rice paddy fields [2]. During the breeding season in Thailand, bettas move to shallow waters to breed [3]. The density in the breeding aggregation was 1.7 fish/m^2^ in a previous study [4]. A study found that domesticated bettas are more aggressive than wild bettas and are well adapted to confinement [5].

Domesticated bettas are known as Siamese fighting fish because of their extreme aggression, which is a consequence of selective breeding for fish fighting. Domesticated bettas have become a popular ornamental fish worldwide because of their bright coloration and the visually appealing long fins of the males. New varieties of bettas with an even more beautiful appearance, such as long, flowing fins and multiple hues, have been produced for competitive shows [1, 6]. When reared as ornamental fish, their aggressiveness can become a serious problem. Although domesticated bettas have been selected for their beautiful appearance, their aggressive nature remains [3]. It is recommended that adult bettas, especially males, should be kept in isolation and remain unaware of the presence of conspecifics as they may become agitated and fight until the other fish dies or is removed from their presence. Although female bettas are generally less aggressive than males and can be kept with conspecifics or other fish species, some females have been reported to display fierce aggressiveness toward cohabiters [7]. These characteristics of bettas can make their care time-consuming and challenging for aquarium keepers. As the demand for ornamental fish is expected to increase, decreasing the aggressiveness of bettas is desirable as it would make them a more suitable species for aquariums as well as during transport. Moreover, the aggressiveness of bettas may lead to stress from social interactions when they are kept with other fish of the same or different species [8]. One solution to this problem is to produce less aggressive bettas by selective breeding. However, this method requires a significant amount of time. An alternative solution is to decrease the aggressiveness of bettas through environmental manipulation.

It is well known that the behavior of animals can be altered by their environment. To enhance animal welfare, a technique called environmental enrichment has been used in zoos and aquariums. Environmental enrichment is defined as the addition of stimuli or provision of choice that results in the improvement of animal well-being [9], and is gradually being applied in farm and laboratory settings [10, 11]. Bloomsmith et al. (1991) identified five major types of enrichment, each of which can be subdivided: social (contact or noncontact), occupational (psychological or exercise), physical (enclosure or accessories), sensory (visual, auditory or other stimuli), and nutritional (delivery or type) [12].

Similar to other animals, teleost fish are expected to undergo numerous behavioral modifications as a result of environmental enrichment, which makes them an ideal model animal for testing relationships between the environment and behavior. Numerous studies revealed that teleost fish can respond to environmental manipulation, particularly during the early stages of life [13, 14].

Spacial comlexity of enclosures during the early rearing period results in behavioral flexibility in various species of teleost. For instance, enriched environments have been shown to stimulate exploratory behavior in hatchery-bred North Sea cod [15], improve foraging behavior in Atlantic salmon [16], and enhance cognitive abilities in *Simochromis pleurospilus* [17]. Enriched environment has also been shown to improve the survival rates of hatchery-reared fish released into the wild [18, 19]. Social environment, cohabitutation with conspecifices, also affect later performance, because social behaviors are partly experience based. Rearing dencity impacted shorling behavior and social learning in guppy [20]. A socially more complex environment together with older group members responded less neophobic toward a novel object in the cooperatively breeding cichlid *Neolamprologus pulcher* [21]. Early social experience determine the courtship patterns of male *Girardinichthys multiradiatus* [22].

It has been reported that aggression in animals is affected by a complex and social environment [23]. However, in teleosts, the results depend on the species or strain of fish [13]. For example, the effects of group size on aggression show different trends in four common species of ornamental fish [24]. A complex environment reduced aggression and even led to cohabitation without fighting in intruder–resident tests with the pearl cichlid *Geophagus brasiliensis* [25], whereas other cichlid species showed opposite results [26]. In male bettas that are socially isolated at 6 weeks after hatching, the incidence of agonistic behaviors increases after the termination of mutual fights [27].

Under general rearing conditions, whether in personal aquariums or in commercial facilities, male bettas become distinguishable from females at 2 to 4 month after hatching and must be separated into individual containers to avoid injuries due to aggression [8, 28]. Females bettas are generally reared in communal tanks, however, some females have been reported to display fierce aggressiveness [7]. Furthermore, it has been documented that a stable community of males and females may be established if sufficient space is available [29]. We hypothesized that the rearing condition affects the aggression level of bettas. In this study, bettas raised in a physically complex and social environment were compared with fish that were relocated to a poor and isolated environment at the juvenile or subadult stage. The aggressive behaviors of the fish were evaluated at the juvenile, subadult, and adult stages. The mirror-image test was used to evaluate aggressive behavior. Although this test has limited predictive value for aggression directed at real opponents [30–32], it was selected because, unlike other behavioral tests, it does not influence the concentrations of sex steroid hormones [33]. Body parameters and plasma concentrations of steroid hormones were measured to evaluate the condition of the fish. The concentrations of estradiol (E_2_) in females and 11-ketotestosterone (11-KT) in males were measured to evaluate sexual maturity, and the cortisol concentration was measured to evaluate the stress response after the mirror-image test.

## Results

### Body parameters

**Fig. 1:**
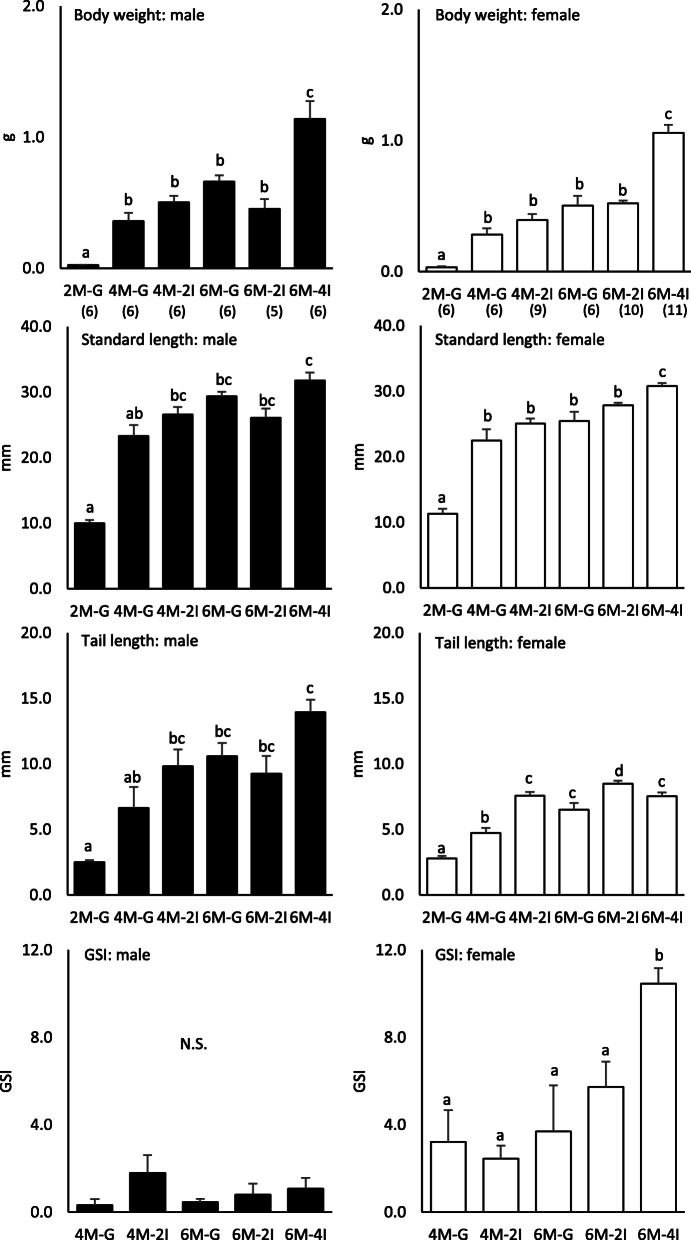
Body conditions of *Betta splendens* subjected to the mirror-image test. Values are expressed as means ± standard errors (SEs) and significant differences are indicated by different letters. N.S., not significant. Statistical significance is defined as *p* < 0.05 based on analysis of variance (ANOVA) followed by the Tukey–Kramer test. Sample sizes are noted in parentheses under the bars in the first graph

Measurements of fish body parameters are shown in Fig. 1. Analysis of variance (ANOVA) revealed significant effects in the experimental group on body weight (F_5,31_ = 27.99, *p* < 0.001 for males; F_5,43_ = 43.42, *p* < 0.001 for females), standard length (F_5,31_ = 53.32, *p* < 0.001 for males; F_5,43_ = 46.19, *p* < 0.001 for females) and tail length (F_5,31_ = 13.05, *p* < 0.001 for males; F_5,43_ = 20.53, *p* < 0.001 for females). Post hoc analysis revealed that among males, 2 M-G individuals had lower body weight, shorter standard length, and shorter tailfins than the other groups. In contrast, 6 M-4I individuals had higher body weight, longer standard length, and longer tail fins than the other groups. Similarly, among females, 2 M-G individuals had lower body weight and shorter standard length, whereas 6 M-4I individuals had higher body weight and longer standard length than the other groups. 2M-G females had shorter tail fins than other groups, whereas 6 M-2I females had the longest tail fins among the experimental groups.

ANOVA also revealed significant differences in gonadosomatic index (GSI) in females (F_4,38_ = 6.54, *p* < 0.001) but not in males (F_4,26_ = 0.838, *p* = 0.514). Post hoc analyses revealed that 6 M-4I females possessed the highest GSI values.

### Behavioral parameters

**Fig. 2:**
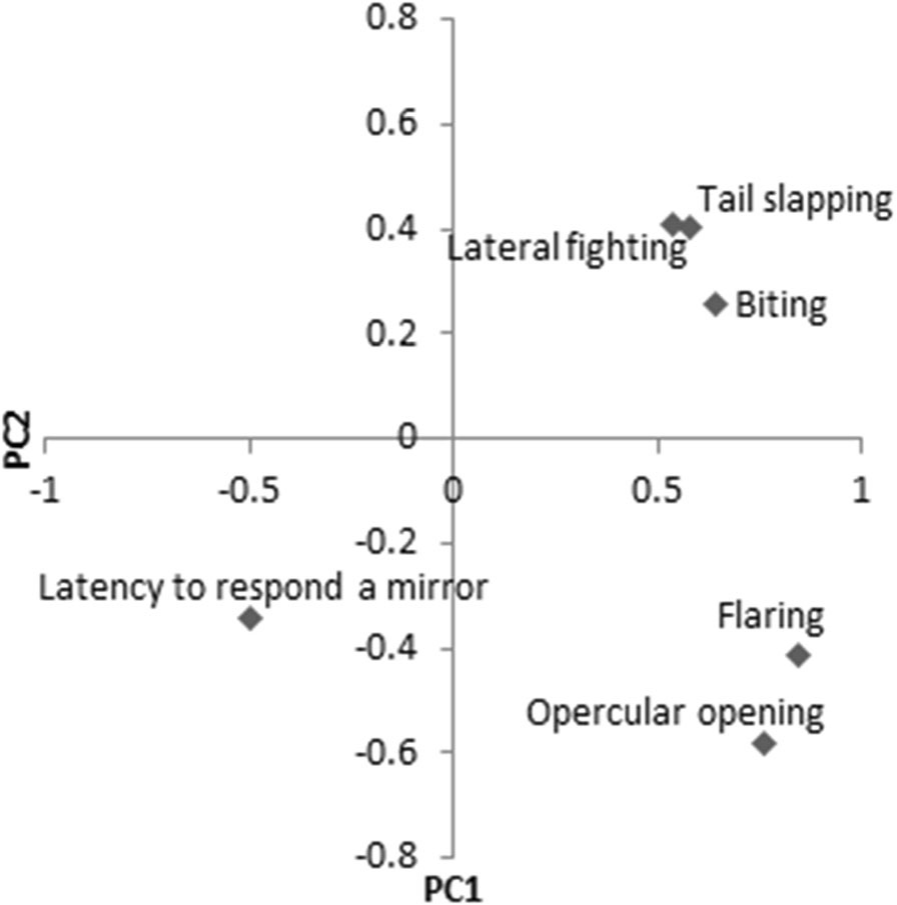
Results of principal component analysis (PCA) with behavioral parameters of *Betta splendens* in response to the mirror-image test according to different rearing environments. Loading plot corresponding to the first two PCs (PC1 and PC2) is shown

**Fig. 3:**
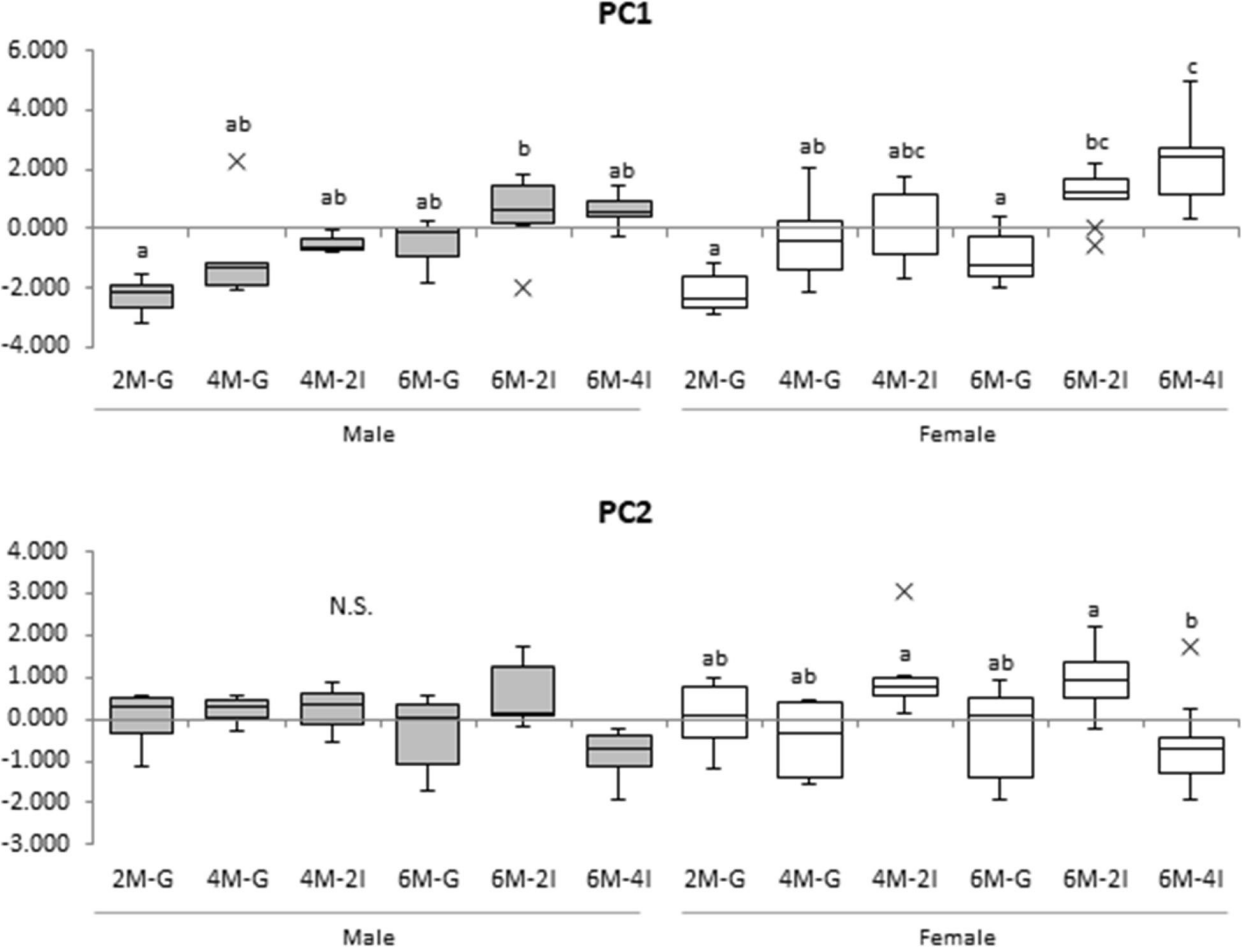
Main component scores of *Betta splendens* in response to the mirror-image test according to different rearing environments.*.* Boxplot values are the median (center line), mean (cross), upper and lower quartiles (upper and lower edges of box), and maximum and minimum values (whiskers). × − marks are outliers. Significant differences between groups are indicated by different letters. N.S., not significant. Statistical significance is defined as *p* < 0.05 based on Kruskal–Wallis test followed by the Steel–Dwass tests

In principal component analysis (PCA), three components explained 70.98% of the variance. The first and second components (PC1 and PC2, respctively) exceeded eigenvalue 1; therefore, PC1 and PC2 were employed for further analysis. Figure 2 shows principal component loadings of each behavioral parameter on a coordinate grid defined by PC1 and PC2. PC1 explained interest in other individuals and latency to respond to a mirror in the negative axis. PC2 explained fighting and threatening, tail slapping, biting, and lateral fighting in the positive axis and opercular opening, flaring, and latency to respond to a mirror in the negative axis. Kruskal–Wallis tests revealed significant differences in main component scores of PC1 in males (*χ*^*2*^ = 17.85, *p* = 0.003) and females (*χ*^*2*^ = 32.42, *p* < 0.001). Significant difference in main component scores of PC2 was observed in females (*χ*^*2*^ = 21.55, *p* < 0.001), but not in males (*χ*^*2*^ = 10.22, *p* = 0.069). In post hoc analysis, 2-MG males showed lower main component scores of PC1 than 6 M-2I males, and 2 M-G and 6 M-G females showed lower main component scores of PC1 than 6 M-2I and 6 M-4I females. 4M-2I and 6M-2I females showed higher main component scores of PC2 than 6 M-4I females (Fig. 3).

The relationship between body parameters and main component scores is shown in Table 1. In males, a statistically significant positive correlation was observed between body parameters and main component scores of PC1 and negative correlation between main component scores of PC 2. In females, statistically significant positive correlations were observed between body parameters and main component scores of PC1. Statisitcally significant negative correlation was observed between female body weitght and main component scores of PC2, but not between total length.

**Table 1:** Correlation coefficient (ρ) between body parameters and main compnent scores of *Betta splendens* subjected to mirror-image tests according to different rearing environment

	Male main component scores				Female main component scores			
	PC1		PC2		PC1		PC2	
	ρ	*p*	ρ	*p*	ρ	*p*	ρ	*p*
Body weight	0.67	<0.001	-0.45	0.009	0.70	<0.001	-0.28	0.047
Total length	0.58	<0.001	-0.41	0.018	0.74	<0.001	-0.11	0.46

### Steroid hormone concentrations

**Fig. 4:**
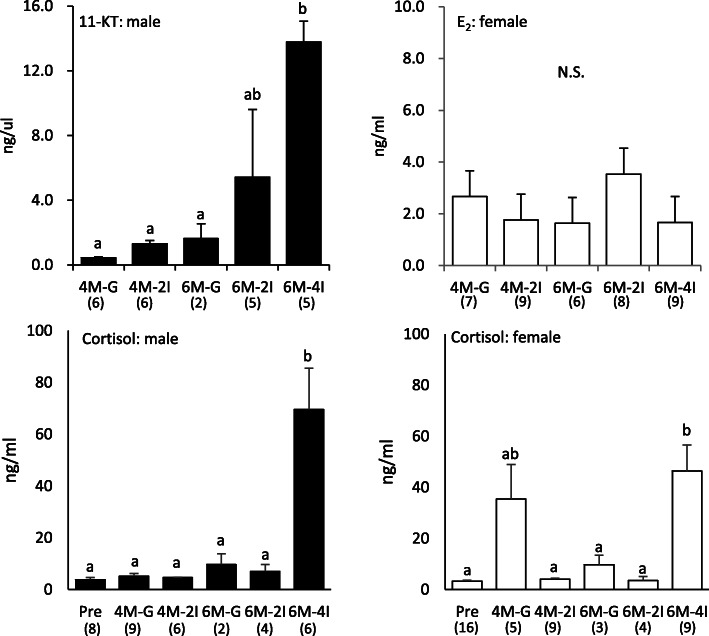
Plasma concentrations of 11-ketotestosterone (11-KT) of males, estradiol (E_2_) of females, and cortisol of both sexes of *Betta splendens* subjected to mirror-image tests according to different rearing environment. Values are means ± standard errors (SEs) and significant differences are indicated by different letters. N.S.: not significant. Statistical significance is defined as *p* < 0.05 based on analysis of variance (ANOVA) followed by the Tukey–Kramer method for 11-KT and E_2_, by the Dunnett’s test for cortisol. Sample sizes are noted in parentheses under the bars in graphs

ANOVA revealed significant differences in male plasma 11-KT concentrations (F_4,23_ = 7.01, *p* = 0.001) but not in female plasma E_2_ concentrations (F_4,41_ = 2.68, *p* = 0.050). 6M-4I males had higher plasma 11-KT concentrations than 4 M-G, 6 M-G, and 4 M-2I males (Fig. 4). Among fish raised in different housing conditions, there were no significant differences in plasma cortisol concentrations of pretest samples (F_2,7_ = 0.28, *p* = 0.76 for males; F_3,15_ = 2.17, *p* = 0.13 for females). In males, ANOVA revealed significant differences in plasma cortisol concentrations (F_5,40_ = 9.40, *p* < 0.001), and post hoc analysis revealed that 6 M-4I individuals had elevated plasma cortisol concentrations after the mirror-image test. No elevations in plasma cortisol concentrations were observed in fish kept in other housing conditions compared with the pretest samples. In females, ANOVA revealed significant differences in plasma cortisol concentrations (F_5,29_ = 14.06, *p* < 0.001). Post hoc analysis revealed that 6 M-4I individuals had elevated plasma cortisol concentrations after the mirror-image test, and no elevations in plasma cortisol concentrations were observed in fish kept in other housing conditions compared with the pretest samples (Fig. 4).

## Discussion

With the increasing popularity of household aquariums, the ornamental fish trade has developed into a multi-billion-dollar industry [34]. Although it has not been a major concern historically, the concept of welfare of ornamental fish is gradually being adopted [35–38]. Therefore, guidelines for the proper housing and transport of ornamental fish must be developed. For bettas, suppressing aggression that arises from their highly territorial nature would contribute to their welfare as ornamental fish. Our study revealed that bettas, regardless of sex, can be housed in groups and display lower levels of aggression if they are housed in groups since hatching under enriched environmental conditions.

PCA revealed that the behaviors of bettas observed during the mirror-image test could be classified into three types. PC1 was negatively associated with “interest in other individuals,” and PC2 was positively associated with “fighting” i.e., tail slapping, biting, and lateral fighting, and negatively associated with “threatening”, i.e., opercular opening and flaring.

The main component score of each individual revealed that the rearing environment affected their agonistic behavior. The main component scores of PC1 increased gradually with increasing age, which reflected the increased regard of other individuals. However, adult bettas respond defferently depending on the rearing environment. Male and female adults isolated as juveniles and female adults isolated subadults had higher scores of PC1, but not adults housed in groups. Adults bettas housed in groups seemed less concerned about the social environment, or had higher social skills. Meanwhile, the main component scores of PC2 remained near zero in housed in groups regardless of their age, which reflected low agonistic interaction or a lack of development of aggression during these stages. Female adults and subadults isolated as juveniles scored higher on PC2, which reflected the increase in actual fighting. Female adults isolated as subadults scored lower, which reflected the increase in threatening behavior. Males showed the same tendency as females on PC2, but statistically significant was not observed, namely, the magnitude of the effect of rearing environment was not obvious in males as that in females.

Ichihashi, Ichikawa and Matsushima [27] reported that isolation at 6 weeks of age promoted highly aggressive behavior in male bettas at 4 months of age, which indicates that earier isolation influence agonistic behaviour of bettas as well as in the present study. Furthermore, our results indicated that the quality of agonisitc behavior displayed by bettas differed according to the timing of isolation. Fish isolated at subadult stage tend to display threatening behaviors, whereas fish isolated at an juvenile stage tend to display fighting behaviours. However, differences were observed between males and females. In males, the environment had low affects on the agonistic behavior compare to females. The result indicates that females respond to environmental manupilation more than males. In general, female bettas are housed in groups because they are known to be less aggressive. However, aggressiveness has been reported in female bettas [7], and a study showed that female bettas can respond to their mirror–image if they are reinforced visually [39]. Together, these results suggest the possibility that female bettas have the ability to display aggression if they are isolated like males because females might respond to environmental factors more flexibly than males.

Aggression has a strong connection with sexual maturity. Particularly in males, a display of aggression is necessary for sexually mature individuals to guard territory, win a mate, and raise offspring [40]. In this study, rearing conditions altered the degree of sexual maturity as well as the display of aggressive behavior. According to male plasma 11-KT concentrations, male tail length, and female GSI, sexual maturity was suppressed in fish housed in groups because group-housed adult fish achieved similar levels of sexual maturity as subadult fish isolated as juveniles. The female plasma E_2_ concentrations did not differ among the experimental groups probably because of the estrus cycle. Surprisingly, fish that achieved sexual maturity were isolated at the subadult stage displayed greater threatening behavior rather than actual fight. Adult bettas isolated at the subadult stage also had better body conditions. The behavior and sexual maturity of fish isolated at the subadult stage might be attributed to differences in nutritional status between isolated and group-housed individuals. In fact, there was a correlation between the body condition and behavior of the fish. However, food intake of the fish in each experimental group was identical, and if nutritional status affected the sexual maturity of fish, the fish that matured most must have been the adult fish isolated at the juvenile stage because these fish could be fed sufficiently for the longest time without competition.

Rearing conditions also affected the stress response of bettas. Cortisol is considered a good indicator of stress in teleosts [41]. In the territorial male convict cichlid *Archocentrus nigrofasciatus*, isolation was shown to induce intense fighting and higher plasma cortisol concentrations after fighting [42]. As mentioned above, hormonal responses to the mirror-image test were induced in adult fish isolated at the subadult stage that displayed greater threatening behavior rather than actual fight. Subadult females housed in groups showed slightly elevated cortisol concentrations, but the change was not statistically significant. This observation may indicate that group housing in an enriched environment and isolation at an earlier life stage can decrease stress responses. However, it is also possible that decreases in the stress response due to chronic stress occurs in group-housed fish [43]. Furthermore, chronic stress might influence sexual maturation of fish [44].

In this study, we compared behavior of bettas either in social and physical enriched environment or in non-social poor environment. Thus, it is difficult to determine whether the social or physical component of the environment was the primary driver of these results. The social environment is known to affect social competence [45]. For example, in *N. pulcher*, individuals that had been reared together with older conspecifics showed more appropriate behaviors depending on social status [46]. *N. pulcher* that were raised in large groups displayed more social behaviors [47]. On the other hand, the complex environment can promote the cognitive ability of fish. Atlantic cod reared in tanks with cobble stones and artificial kelp on the bottom was more adept at learning how to forage on novel prey than those reared in bare tanks [48]. Habitat enrichment improved navigation skills in Atlantic salmon [49]. In the present study, both complex and social environment interacting together might led to decreased aggressive behavior in bettas. In social environments, bettas may learn how to interact with each other appropriately, and bettas can promote the cognitive ability in complexed environments and learn to avoid agonistic interactions.

The question remains why the timing of relocation from social and complexed environment to isolated poor environment altered the level of aggression and why sexual maturation was suppressed in fish that were relocated at the juvenile stage. Juvenile European eels (*Anguilla anguilla*) that are not highly gregarious display higher than expected aggressiveness toward other fish [50]. Earlier isolation might lead to less experience with social interactions and results in elevated levels of aggressiveness [51]. The betta is a solitary and highly territorial species, and the influence of social interaction on their behavior is poorly understood [4]. Interaction between individuals at early stages of life might be essential for proper development, including behavioral and sexual development, even in nonsocial species such as bettas. If sexual maturity is considered a suitable indicator of welfare [52], bettas isolated at the subadult stage might display proper development. To confirm this hypothesis, a detailed study of the natural history of wild bettas, especially regarding their distribution and the dispersal pattern of larval to subadult individuals, is needed.

## Conclusions

In this study, the timing of isolation altered the level of aggressive behavior; earlier isolation into poor environment increased finghting behavior and later increased threatening behavior. Female bettas might respond to environmental factors more flexibly than males. Group housing in an enriched environment successfully decreased aggressive behavior in adult bettas. Adult bettas housed in groups in enriched environment displayed the same level of agonistic behavior as juvenile fish. If a suitable environment is available, both males and females could be housed in groups without displaying excessive aggression. However, in this study, group housing in enriched environment suppressed sexual maturation. Bettas that were socially isolated into poor environment at the juvenile stage also exhibited suppressed sexual maturation, whereas fish isolated into poor environment at the subadult stage achieved sexual maturation. Judging from their level of sexual maturity, bettas isolated as subadults with increased threatening behavior show proper development.

## Methods

### Experimental animals

Three pairs of sexually mature bettas (traditional type) were purchased from a local distributor (Sugano Pet Fish Shop, Iwaki City, Fukushima, Japan) and bred in the laboratory. The pairs originated from the same breeder, but it was unknown whether they were genetically related. The larvae were reared in groups of 12 to 30 individuals per tank (30 × 20 × 20 cm). After the larvae grew in size and were able to tolerate water flow, each tank was equipped with a closed filtration system. The fish were fed brine shrimp every day during the larval and juvenile stages and commercial pellets (Hikari-Betta, Kyorin Co., Ltd., Hyougo, Japan) at later life stages. The food intake levels of individual fish were kept as close to equal as possible. The fish were maintained at a water temperature of 25 °C–26 °C under natural light conditions. All protocols described below were approved by the Experimental Animal Care Committee at Iwaki Meisei University and followed Iwaki Meisei University’s Policies Governing the Use of Live Vertebrate Animals and the Japan Ethological Society’s Guidelines for Research on Animal Behaviour.

### Experimental design

The juveniles were reared in groups under enriched environmental conditions for 2 months after hatching (60 days post hatch [dph]). Under the group-rearing condition, the fish were housed in groups of 12 to 30 individuals in 30 × 20 × 20-cm tanks with water plants (*Egeria densa*), rocks, and shelters. The number of individuals varied because the mortality rate of the larvae varied among the tanks. At the age of 2 months, 40 fish were relocated to an isolated, poor environment. The isolation tanks measured 10 × 10 × 10 cm and were composed of opaque plastic that prevented the fish from seeing each other. A 10-cm stalk of *E. densa* was provided in each isolation tank instead of a water filtration system. At this time point, 12 group-housed fish were subjected to a mirror-image test. At the age of 4 months (120 dph), the procedure was repeated; 20 fish were transferred from the group-housed environment to the isolated environment, and behavioral testing was conducted for both group-housed fish and fish isolated at 2 months of age. At the age of 6 months (180 dph), mirror-image tests were conducted on fish from all rearing conditions. The following abbreviations for the experimental groups are used in the text and figures: 2 M-G, fish raised in a group until 2 months of age; 4 M-G, fish raised in a group until 4 months of age; 6 M-G, fish raised in a group until 6 months of age; 4 M-2I, fish raised in a group until 2 months of age and then isolated until 4 months of age; 6 M-2I, fish raised in a group until 2 months of age and then isolated until 6 months of age; 6 M-4I, fish raised in a group until 4 months of age and then isolated until 6 months of age.

The sample size and sex composition of the experimental groups varied because it is difficult to determine the sex of bettas at 2 months of age without dissection. Under our laboratory rearing conditions, the age groups were defined as follows: 2-month-old fish were considered sexually immature juveniles, 4-month-old fish were considered subadults at the onset of sexual maturation, and 6-month-old fish were considered sexually mature adults. The experimental fish were selected from each clutch equally to avoid unexpected influences on the data.

### Mirror-image test

Fish were randomly assigned to experimental groups and acclimated for 10 min in a 20 × 10 × 10-cm polycarbonate tank, after which they were presented with a 9 × 8-cm mirror for 10 min. We recorded the duration (in seconds) of flaring and opercular opening display to evaluate threatening behavior, lateral fighting, the frequencies of tail slapping and biting to evaluate fighting behavior. We also recorded the latency (in seconds) to respond to the mirror. Descriptions of the behavioral parameters are given in Table 2. All tests were performed between 12:00 and 18:00. Individual fish were tested only once and euthanized after mirror-image test for further examinations.

**Table 2:** Descriptions of behavioral parameters of *Betta splendens* subjected to the mirror-image test according to different rearing environments

Behavioural elements	Discription
Latency to respond to a mirror	Latency of time (s) of focal individual to locate or approach mirror.
Duration of lateral fighting	Duration of time (s) of focal individual to display itself laterally to a mirror for comparing its body size to self-image in a mirror.
Duration of flaring	Duration of time (s) of focal individual to spread its fins as a threat display.
Duration of opercular opening	Duration of time (s) of focal individual to display its opercular opening as a threat display.
Frequency of tail slapping	Frequency of focal individual slapping its tail to its self-image in a mirror.
Frequency of biting	Frequency of focal individual biting its self-image in a mirror.

### Body parameters and steroid hormone concentrations

Immediately after the termination of the mirror-image test, the fish were captured and euthanized with 300 ppm of MS222 (tricaine methanesulfonate; Sigma-Aldrich, St. Louis, MO, USA). The fish were captured by catching them in the water using hands to avoid harming them, and they were moved to a smaller tank with the dissolved anesthetic chemical. To avoid any influence on serum cortisol concentrations, anesthesia was performed within 3 min of capture. Total body length, standard length, body weight, and gonad weight were measured. The standard length was subtracted from the total body length to obtain the tail length. The body and gonad weights were used to calculate GSI (GSI = gonad weight / body weight × 100) to evaluate sexual maturity. The sex of 2-month-old fish was determined after dissection using a stereomicroscope, but GSI was not calculated because the gonads were too small to weigh. Blood was collected from the caudal vessel using heparinized capillary tubes, and blood samples was immediately centrifuged. Plasma was separated and stored in plastic tubes at − 20 °C until assay.

### Hormone assays

Because the number of pretest plasma samples was small in each group, the plasma cortisol concentrations of fish that did not undergo the mirror-image test (pretest) were combined and evaluated. Five microliters of plasma was extracted using 2 mL of diethyl ether and resuspended in 500 μL of enzyme immunoassay (EIA) buffer (Cayman Chemical, Ann Arbor, MI, USA). The concentrations of the hormones were measured using a commercially available EIA kit (Cayman Chemical) following the manufacturer’s instructions. All samples were measured in triplicate. The cross-reactivity of antibodies is shown in Table 3. The inter- and intra-assay coefficients of variation were 14.7 and 7.3% for E_2_, 8.2 and 8.3% for 11-KT, and 7.6 and 8.4% for cortisol, respectively.

**Table 3:** Cross-reactivity of antibodies used for enzyme immune assay of plasma estradiol (E_2_), 11-ketotestosterone (2 11-KT), and cortisol

estradiol		11-ketotestosterone		cortisol	
Compound	Cross reactivity		Cross reactivity		Cross reactivity
estradiol	100%	11-ketotestosterone	100%	cortisol	100%
estorone	12%	adrenosterone	2.9%	corticosoterone	0.14%
estriol	0.30%	4-androstan-11β, 17β-diol- 3-one	0.01%	cortisone	0.13%
androstendiol	0.02%	5-androstan-17β-ol- 3-one	<0.01 %	androstendione	<0.01 %
testosterone	<0.01 %	testosterone	<0.01 %	testosterone	<0.01 %

### Statistical analysis

Statistical analyses were performed using BellCurve for Excel version 3.21 (Social Survey Research Information Co., Ltd., Tokyo, Japan). A probability level (*p*) of < 0.05 was considered to indicate statistical significance. All parameters were analyzed separately for males and females. Body parameters and steroid hormone concentrations were compared using one-way ANOVA followed by the Tukey–Kramer multiple comparison test. Plasma cortisol concentrations were compared using one-way ANOVA followed by Dunnett’s test. All parameters analyzed by ANOVA met the assumptions for normal distribution. For behavioral parameters, PCA was performed, and the main component scores of each individual were compared using the Kruskal–Wallis test followed by Steel–Dwass multiple comparison post hoc tests. The relation between the body and behavioral elements was investigated by Pearson’s correlation coefficient test.

## Data Availability

The datasets used and analyzed during the current study are available from the corresponding author on reasonable request.

## Abbreviations

ANOVA: Analysis of variance
EIA: Enzyme immunoassay
GSI: Gonadosomatic index (gonad weight / body weight × 100)
DPH: Days post hatching
11-KT: 11-ketotestosterone

## References

[1] Monvises A, Nuangsaeng B, Sriwattanarothai N, Panijpan B (2009). The Siamese fighting fish: well-known generally but little-known scientifically. Scienceasia..

[2] Gordon M, Axelrod HR (1968). Siamese fighting fish.

[3] Watson C, DiMaggio M, Hill J, Tuckett Q, Yanong R (2019). Evolution, culture, and care for *Betta splendens*. EDIS..

[4] Jaroensutasinee M, Jaroensutansinee K (2001). Bubble nest habitat characteristics of wild Siamese fighting fish. J Fish Biol.

[5] Verbeek P, Iwamoto T, Murakami N (2008). Variable stress-responsiveness in wild type and domesticated fighting fish. Physiol Behav.

[6] Panijpan B, Sriwattanarothai N, Kowasupat C, Ruenwongsa P, Jeenthong T, Phumchoosri A (2017). Biodiversity of bubble-nest building and mouth-brooding fightingfish species of the genus betta in Southeast Asia. Thai Nat Hist Mus.

[7] Braddock JC, Braddock ZI (1955). Aggressive behavior among females of the Siamese fighting fish, *Betta splendens*. Physiol Zool.

[8] Pleeging CCF, Moons CPH (2017). Potential welfare issues of the Siamese fighting fish (*Betta splendens*) at the retailer and in the hobbyist aquarium. Vlaams Diergen Tijds.

[9] Shepherdson DJ, Mace G, Olney P, Feistner ATC (1994). The role of environmental enrichment in the captive breeding and reintroduction of endangered species. Creative conservation: interactive management of wild and captive animals.

[10] Olsson IAS, Dahlborn K (2002). Improving housing conditions for laboratory mice: a review of environmental enrichment. Lab Anim.

[11] Boissy A, Lee C (2014). How assessing relationships between emotions and cognition can improve farm animal welfare. Rev Sci Tech.

[12] Bloomsmith MA, Brent LY, Schapiro SJ (1991). Guidelines for developing and managing an environmental enrichment program for nonhuman-primates. Lab Anim Sci.

[13] Näslund J, Johnsson JI (2016). Environmental enrichment for fish in captive environments: effects of physical structures and substrates. Fish Fish.

[14] Jonsson B, Jonsson N (2014). Early environment influences later performance in fishes. J Fish Biol.

[15] Braithwaite VA, Salvanes AGV (2005). Environmental variability in the early rearing environment generates behaviourally flexible cod: implications for rehabilitating wild populations. Proc Biol Sci.

[16] Brown C, Davidson T, Laland K (2003). Environmental enrichment and prior experience of live prey improve foraging behaviour in hatchery-reared Atlantic salmon. J Fish Biol.

[17] Kotrschal A, Taborsky B (2010). Environmental change enhances cognitive abilities in fish. PLoS Biol.

[18] Brown C, Day RL (2002). The future of stock enhancements: lessons for hatchery practice from conservation biology. Fish Fish.

[19] Rosengren M, Kvingedal E, Näslund J, Johnsson JI, Sundell K (2017). Born to be wild: effects of rearing density and environmental enrichment on stress, welfare, and smolt migration in hatchery-reared Atlantic salmon. Can J Fish Aquat Sci.

[20] Chapman BB, Ward AJW, Krause J (2008). Schooling and learning: early social environment predicts social learning ability in the guppy, *Poecilia reticulata*. Anim Behav.

[21] Bannier F, Tebbich S, Taborsky B (2017). Early experience affects learning performance and neophobia in a cooperatively breeding cichlid. Ethology..

[22] de Gasperin O, Garcia CM (2014). Congenital predispositions and early social experience determine the courtship patterns of males of the Amarillo fish. Behav Ecol Sociobiol.

[23] Van Loo PLP, Van Zutphen LFM, Baumans V (2003). Male management: coping with aggression problems in male laboratory mice. Lab Anim.

[24] Saxby A, Adams L, Snellgrove D, Wilson RW, Sloman KA (2010). The effect of group size on the behaviour and welfare of four fish species commonly kept in home aquaria. Appl Anim Behav Sci.

[25] Kadry VO, Barreto RE (2010). Environmental enrichment reduces aggression of pearl cichlid, *Geophagus brasiliensis*, during resident-intruder interactions. Neotrop Ichthyol.

[26] Nijman V, Heuts BA (2011). Aggression and dominance in cichlids in resident-intruder tests: the role of environmental enrichment. Neotrop Ichthyol.

[27] Ichihashi T, Ichikawa Y, Matsushima T (2004). A non-social and isolate rearing condition induces an irreversible shift toward continued fights in the male fighting fish (*Betta splendens*). Zool Sci.

[28] Goldstein R (2004). The betta handbook.

[29] Goldstein SR (1975). Observations on the establishment of a stable community of adult male and female siamese fighting fish (*Betta splendens*). Anim Behav.

[30] Desjardins JK, Fernald RD (2010). What do fish make of mirror images?. Biol Lett.

[31] Arnott G, Beattie E, Elwood RW (2016). To breathe or fight? Siamese fighting fish differ when facing a real opponent or mirror image. Behav Process.

[32] Balzarini V, Taborsky M, Wanner S, Koch F, Frommen JG (2014). Mirror, mirror on the wall: the predictive value of mirror tests for measuring aggression in fish. Behav Ecol Sociobiol.

[33] Oliveira RF, Carneiro LA, Canário AVM (2005). Behavioural endocrinology: no hormonal response in tied fights. Nature..

[34] Dey VK (2016). The global trade in ornamental fish. Infofish Int.

[35] Brown C, Dorey C (2019). Pain and emotion in fishes—fish welfare implications for fisheries and aquaculture. Anim Stud.

[36] Huntingford FA, Adams C, Braithwaite VA, Kadri S, Pottinger TG, Sandøe P (2006). Current issues in fish welfare. J Fish Biol.

[37] Sneddon LU, Lopez-Luna J, Wolfenden DCC, Leach MC, Valentim AM, Steenbergen PJ, et al. Fish sentience denial: muddying the waters. Anim Sentience. 2018;21 https://animalstudiesrepository.org/animsent/vol3/iss21/1/.

[38] Stevens CH, Croft DP, Paull GC, Tyler CR (2017). Stress and welfare in ornamental fishes: what can be learned from aquaculture?. J Fish Biol.

[39] Elcoro M, da Silva SP, Lattal KA (2008). Visual reinforcement in the female Siamese fighting fish, *Betta splendens*. J Exp Anal Behav.

[40] Svare BB (1983). Hormones and aggressive behaviour.

[41] Pottinger TG, Branson EJ (2008). The stress response in fish-mechanisms, effects and measurement. Fish welfare 2008 19-31.

[42] Earley RL, Edwards JT, Aseem O, Felton K, Blumer LS, Karom M (2006). Social interactions tune aggression and stress responsiveness in a territorial cichlid fish (*Archocentrus nigrofasciatus*). Physiol Behav.

[43] Mommsen TP, Vijayan MM, Moon TW (1999). Cortisol in teleost: dynamics, mechanisms of action, and metabolic regulation. Fish Biol Fish.

[44] Schreck CB (2010). Stress and fish reproduction: the roles of allostasis and hormesis. Gen Comp Endocrinol.

[45] Taborsky B, Oliveira RF (2012). Social competence: an evolutionary approach. Trends Ecol Evol.

[46] Taborsky B, Arnold C, Junker J, Tschopp A (2012). The early social environment affects social competence in a cooperative breeder. Anim Behav.

[47] Fischer S, Bessert-Nettelbeck M, Kotrschal A, Taborsky B (2015). Rearing-group size determines social competence and brain structure in a cooperatively breeding cichlid. Am Nat.

[48] Strand DA, Utne-Palm AC, Jakobsen PJ, Braithwaite VA, Jensen KH, Salvanes AGV (2010). Enrichment promotes learning in fish. Mar Ecol Prog Ser.

[49] Salvanes AGV, Moberg O, Ebbeson LOE, Nilsen TO, Jensen KH, Braithwaite VA (2013). Environmental enrichment promotes neural plasticity and cognitive ability in fish. Proc R Soc B.

[50] Geffroy B, Bru N, Dossou-Gbété S, Tentelier C, Bardonnet A (2014). The link between social network density and rank-order consistency of aggressiveness in juvenile eels. Behav Ecol Sociobiol.

[51] Stevenson PA, Rillich J (2013). Isolation associated aggression-a consequence of recovery from defeat in a territorial animal. PLoS One.

[52] Oppedal F, Lasse Taranger GL, Juell JE, Fosseidengen JE, Hansen T (1997). Light intensity affects growth and sexual maturation of Atlantic salmon (*Salmo salar*) postsmolts in sea cages. Aquat Living Resour.

